# Response to Re: Estimating and reducing dose received by cardiac devices for patients undergoing radiotherapy

**DOI:** 10.1120/jacmp.v17i4.6510

**Published:** 2016-07-08

**Authors:** Louis Archambault, Nicolas Varfalvy, Alexandra Bourgouin

**Affiliations:** ^1^ Department of Radiation Oncology Centre Hospitalier Universitaire de Quebec Quebec City QC Canada

To the Editor:

We have read the comments^(1)^ made regarding our recent manuscript(2) and we welcome the chance to address some of the issues raised in that letter to the editor. First and foremost, we would like to clarify that our manuscript does not intend to recommend the widespread use of lead shielding as protection for cardiac implanted electronic devices (CIEDs). Our goal was twofold. First we wanted to present a simple model to estimate out‐of‐field doses and use this model to assess doses that can be delivered to CIEDs. Second we wanted to quantify possible dose reductions resulting from the use of a lead shielding. While dose reductions were indeed observed, they may not always be significant.

There is no doubt that the model we used must be a function of the radial distance. Equation (1) ^(2)^ was only presented to show the general form of the exponential model used. The dependence with the radial distance was only explicit in Eq. (2),^(2)^ which is:

(1) $$D=N_{MU}(\alpha e^{-d/\beta }+\gamma )$$

where *d* is the radial out‐of‐field distance. Thus Eq. (2) is the same as the one proposed by Mihailidis.^(1)^

As pointed out,^(1)^ most measurements involved in our work were taken in conditions that are unusual for clinical measurements: out‐of‐field and low doses at shallow depths. Such measurements can be challenging for most detectors and require careful considerations. The excellent linearity and good energy independence of a plastic scintillation detector (PSD)^(3–6)^are actually the main reasons that motivated the use of a PSD over more common detector types, such as silicon diodes that overrespond to low photon energies due to the higher interaction probability per unit mass of Silicon compared to water. PSDs like the W1 have been shown to be water equivalent over a large range of energy, including radiological energies,^(7)^ which makes it ideal for out‐of‐field measurements.

Furthermore, side‐by‐side comparison between the Exradin W1 and ion chambers (an IBA CC04 and a Exradin A11 parallel plate chamber) for out‐of‐field measurements were also performed, and a manuscript on that topic is currently in press.^(8)^ Measurements at a distance of 3 cm outside a $15\times 15\;{\text{cm}}^{2}$ field made with both the W1 and the CC04 agreed to within 5% for depths greater than 1.5 cm for 6 MV and 23 MV beams. At shallower depths, the dose gradient varied more rapidly and discrepancies up to 10% were observed. In these cases the poorer spatial resolution of the ion chamber could explain part of the observed differences. Comparison between a parallel plate ion chamber and the W1 agreed to within 0.2 and 0.1 cGy for doses of 0.5 cGy for photon and electron beams, respectively. These discrepancies were within the estimated statistical uncertainties. Having been able to measure small surface doses out of the primary radiation field with a detector calibrated using the manufacturer's recommended procedure is, in our opinion, a testimony of the great versatility of PSD detectors. When comparing doses between 6 MV and 23 MV it is important to recall that our measurements were taken at a depth of 1.5 cm (the average depth of CIED at our institution), which is shallower than other published data. For example, Fig. 18 of TG‐36^(9)^ presents a comparison between energies measured at 10 cm depth. It is thus difficult to compare measurements at such different depths. Nevertheless, when looking at posterior‐anterior (PA) beams that are necessarily made at a much greater depths, our measurements are indeed relatively independent of beam energy.

Our observations at shallow depths appear to be in line with other surface dose measurements close to the field edges.^10^, ^11^

It is important to stress again that the point of our manuscript is not to propose or recommend the use of lead shielding as a perfect solution to protect CIEDs, but instead to show that such shielding is a simple and inexpensive method of reducing doses to CIED. We agree that results can greatly vary depending on the linac type and the geometry of its head. Linacs from other manufacturers will likely show a different behavior. Nevertheless, the simple model we propose could be fitted to any data and help estimate dose to CIED in regions where treatment planning systems are well known to offer suboptimal dose calculation accuracy. In that regard, Fig. 2^(2)^ should be seen as a demonstration that our dose estimation model can work in different situations, rather than a recommendation to use lead shielding. In our clinic, lead covered in thermoplastic was used because it is easy to manipulate and position on the patient, but we agree that other materials could be used to the same effect.

There is mounting evidences that the presence of neutrons is strongly correlated with CIED events during radiation therapy,^(12)^ but CIED events are also observed in patients treated with fields that do not produce neutrons. Therefore, adding some shielding that can reduce the dose received by CIEDs is well aligned with the ALARA (i.e., as low as reasonably achievable) principle of radiation protection. Nevertheless, the pertinence of using a shielding when treating CIED should be evaluated carefully by any clinic.

## COPYRIGHT

This work is licensed under a Creative Commons Attribution 3.0 Unported License.
